# The complicated management of a patient following transarterial chemoembolization for metastatic carcinoid

**DOI:** 10.1186/1477-7819-6-125

**Published:** 2008-11-25

**Authors:** Andrew C Pearson, Steven Steinberg, Manisha H Shah, Mark Bloomston

**Affiliations:** 1 Department of Surgery, Doctors' Hospital West, Columbus, Ohio, USA; 2 Department of Surgery, Ohio State University Medical Center, Columbus, Ohio, USA; 3 Division of Hematology and Oncology, Ohio State University Medical Center, Columbus, Ohio, USA

## Abstract

**Background:**

Transarterial Chemoembolization (TACE) has been recognized as a successful way of managing symptomatic and/or progressive hepatic carcinoid metastases not amenable to surgical resection. Although it is a fairly safe procedure, it is not without its complications.

**Case presentation:**

This is a case of a 53 year-old woman with a patent foramen ovale (PFO) and mild pulmonary hypertension who underwent TACE for progressive carcinoid liver metastases. She developed acute heart failure, due to a severe inflammatory response; this resulted in pneumatosis intestinalis due to non-occlusive mesenteric ischemia. We describe the successful non-operative management of her pneumatosis intestinalis and the role of a PFO in this patient's heart failure.

**Conclusion:**

TACE remains an effective and safe treatment for metastatic carcinoid not amenable to resection, this case illustrates the complexity of complications that can arise. A multi-disciplinary approach including ready access to advanced critical care facilities is recommended in managing such complex patients.

## Case presentation

A 53 year-old woman reported progressive diarrhea, flushing, and weight loss over several years. Her medical history was significant for hypertension and seizure disorder. In December of 2006, she underwent a CT scan of the abdomen as part of a workup for abdominal pain; she was found to have a large mass in the left lobe of the liver. A biopsy was obtained which demonstrated metastatic well differentiated neuroendocrine carcinoma. Follow-up colonoscopy showed a 2.5 cm mass in her terminal ileum. Somatostatin receptor scintigraphy showed marked bilobar hepatic uptake consistent with metastatic carcinoid but no extrahepatic metastatic disease.

In March 2007, she underwent a right hemicolectomy to remove the presumed primary lesion. Intraoperatively, her hepatic disease was felt to be too extensive for resection. Pathology showed a 3.2 cm well-differentiated neuroendocrine carcinoma of the terminal ileum with lymphatic and vascular invasion, and 8/25 lymph nodes tested positive for metastatic disease. She was started on long acting somatostatin analog therapy post-operatively, which controlled her symptoms of flushing and diarrhea.

After her exploration, she developed post-operative hypoxia necessitating a transthoracic echocardiogram shortly after surgery. The echocardiogram showed normal left ventricular systolic function and severe tricuspid regurgitation. Heart catheterization demonstrated significantly elevated right atrial pressures and a patent foramen ovale (PFO). The foramen ovale was temporarily occluded with a 7-French balloon, and her oxygen saturation increased from 88% to 99%, confirming the presence of a severe right to left atrial shunt. She experienced a drop in cardiac output; therefore, a permanent solution was not sought.

In July 2007, she was found to have progressive hepatic metastases after being referred to the Neuroendocrine Tumor Clinic at Ohio State University for further management. Transarterial Chemoembolization (TACE) was recommended and a vena cava filter was placed to prevent a paradoxical embolus during her post-procedure convalescence. Whole liver TACE was undertaken in August 2007 with Cisplatin AQ 50 mg, Doxorubicin 30 mg, Mitomycin 20 mg, Iodixanol 3200 mg, and 300–500 and 500–700 micron embospheres. As per institutional protocol, somatostatin analog (octreotide) was continuously infused before, during, and after TACE.

In the first 12 hours following TACE, the patient had two seizures and mental status changes. Brain imaging did not demonstrate acute changes so the patient was treated for encephalopathy. Over the ensuing 24 hours, she became progressively more somnolent and developed worsening abdominal tenderness. She was transferred to the intensive care unit and intubated for airway protection. Once placed on positive pressure ventilation, she became hypotensive and hypoxic, necessitating large volume resuscitation and vasopressor therapy. Her hypoxia was unresponsive to increases in oxygen supplementation and positive end expiratory pressure (PEEP). Pulmonary artery catheter measurement demonstrated moderate pulmonary hypertension with pulmonary artery pressures as high as 70 mmHg and depressed cardiac output of 3–3.5 liters per minute. During this time, she developed abdominal tenderness.

**Figure 1:**
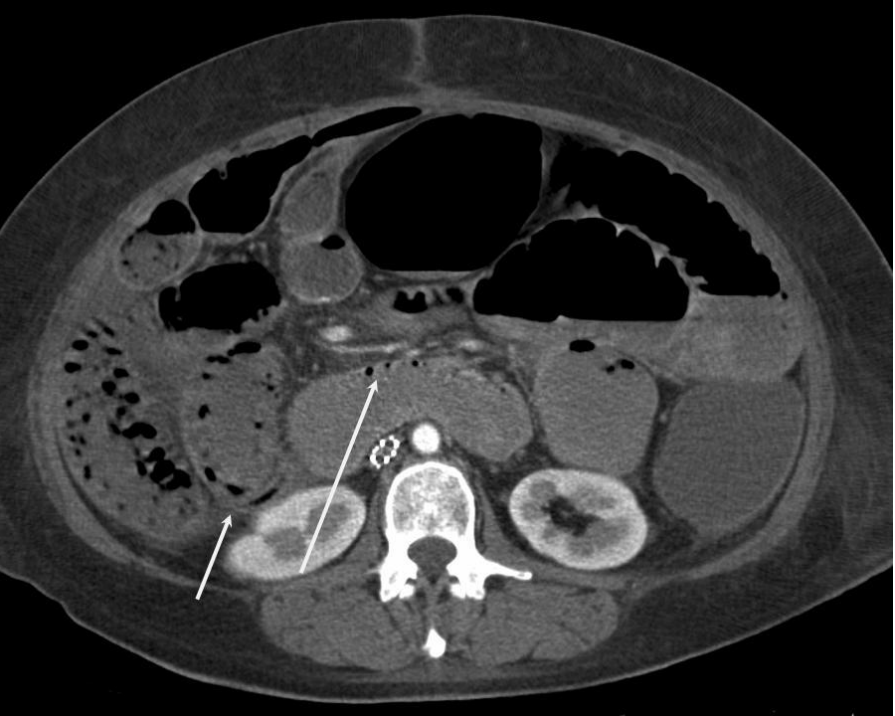
Computed tomography demonstrating pneumatosis intestinalis within the walls of the small and large bowel (arrows).

Computed tomography (CT) scan demonstrated pneumatosis intestinalis involving the small bowel without evidence of perforation (Figure 1). At that time, her abdominal examination was benign; she showed no systemic signs of infection, including negative cultures from blood, urine, and sputum. Broad spectrum antibiotics were started, and she was kept on bowel rest.

Echocardiogram demonstrated pulmonary hypertension, severe right-to-left shunting across her PFO and left ventricular ejection fraction of 35% (compared to 65% pre-TACE). Efforts were made to minimize her PEEP and accept lower arterial oxygen saturations of 85 to 88%. As the acute inflammatory response abated over the next 72 hours, the patient's mental status cleared and her abdominal pain resolved. She rapidly weaned from the ventilator and tolerated enteral feeding. She was ultimately discharged to home 10 days after her TACE without residual sequelae.

**Figure 2:**
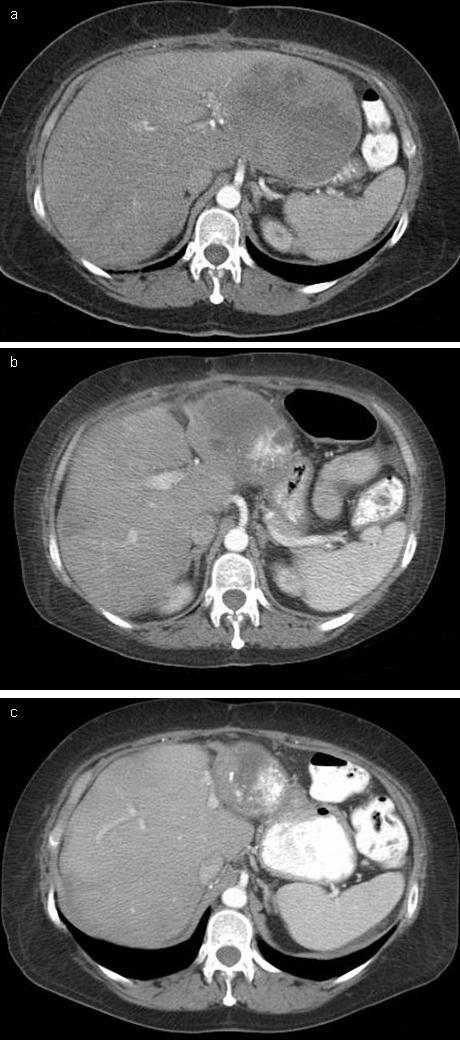
Computed tomography scan of metastatic carcinoid prior to TACE (A), four months after TACE (B), and eight months after TACE (C) showed marked reduction in hepatic tumor burden.

After discharge, the patient completely recovered and had significant serologic, radiographic, and symptomatic response to TACE. At eight month follow-up, the patient showed marked reduction in hepatic tumor burden (Figure 2) and near-total resolution of her carcinoid syndrome symptoms. Her serum pancreastatin levels decreased from 13,400 pg/mL (normal <135 pg/mL) prior to TACE to 1,230 pg/mL. She has undergone subsequent echocardiography with improvement in her pulmonary hypertension and restoration of a normal ejection fraction.

## Discussion

This patient's complicated course illustrates the complexity of patients with advanced carcinoid and the challenges that can be faced following TACE. Our discussion will focus on the role her PFO played in her ventilator management, as well as the non-operative management of pneumatosis intestinalis.

Patent foramen ovale is found in approximately 25% of the population [1]. The majority of the time, this congenital heart anomaly is clinically silent [2]. Manifestations of clinically significant PFO's include: paradoxical embolism, orthostatic desaturation in the setting of platypnea-orthodeoxia syndrome (in the presence of a PFO, a right to left shunt results from redirection of inferior vena caval flow toward the atrial septum upon standing, resulting in postural hypoxemia), neurological decompression illness in divers, migraine headache with aura, and refractory hypoxemia in a certain subset of patients [1,3]. Hypoxemia in the setting of a PFO without pulmonary hypertension is rare, but has been reported in cases of pulmonic stenosis, pulmonary fibrosis, tricuspid regurgitation, hypoplastic right ventricle, right ventricular infarction, adult respiratory distress syndrome, altered right ventricular compliance, and following right pneumonectomy [4]. The most common factors responsible for enhancing a right to left shunt through a PFO (and thus new onset hypoxemia) are positive pressure mechanical ventilation with high positive end expiratory pressure and cardiac tamponade [5].

Transarterial chemoembolization for metastatic carcinoid is commonly associated with fevers, pain, leukocytosis, nausea, malaise, and fatigue [6,7]. This so-called *postchemoembolization syndrome *emphasizes the inflammatory response associated with TACE, perhaps due to tumor lysis. While these findings are typically managed on an outpatient basis, they can initiate a cascade of events as seen in the patient described herein that can prove life threatening. More severe inflammatory reactions TACE can occasionally be attributed to an intratumoral arterio-venous fistula. In this situation, the chemotherapeutic mixture would flow through the tumor and directly into the pulmonary circulation. Although the pre-TACE angiogram in this patient did not reveal obvious shunting, had the chemotherapeutic/particle mixture traveled to her pulmonary circulation, we could expect her right heart pressures to increase, exacerbating a right to left shunt. Additionally, this right to left intracardiac shunt would have allowed the systemic circulation of the chemotherapeutic/particle mixture, initiating a systemic inflammatory response.

With respect to the case presented, her PFO played a crucial part in her complicated course. Rapid rise in her pulmonary artery pressures, presumptively secondary to the inflammatory response, exacerbated her right-to-left shunt, resulting in progressive refractory hypoxemia. Her condition was further worsened by positive pressure ventilation and PEEP causing marked reduction in cardiac output and end-organ hypoperfusion. This was evident by somnolence, oliguria, and pneumatosis intestinalis.

Because roughly one quarter of the population has a potentially patent foramen ovale, interatrial right to left shunting may occur more frequently than is currently recognized. When considering TACE in patients with a history of PFO or an abnormal heart murmur, thorough cardiac investigation should be sought. Carcinoid heart disease occurs in half of patients with metastatic carcinoid tumors, and usually manifests as thickening and incompetence of the right heart valves [8]. Less commonly, the left side of the heart can be effected by carcinoid heart disease. In this situation, PFO represents the major etiologic factor [9]. In 20% of patients with a carcinoid tumor, the initial manifestation is due to cardiac complications. A prospective study by Mansencal, et al [9] showed that percutaneous closure of PFO in patients with symptomatic carcinoid heart disease improved New York Heart Association functional status, 6-minute walking distance, and arterial blood gas results. Additionally, a case report by Chaudhari, et al. demonstrated the symptomatic relief of left-sided carcinoid heart disease following percutaneous closure of PFO [10]. Although these interventions are largely providing symptomatic relief, they do appear to be improving the quality of life in this select group of patients.

The management of pneumatosis intestinalis in this patient also proved quite challenging. Given the timing of onset after evidence of systemic hypoperfusion and the lack of evidence of sepsis, we elected to manage her non-operatively, as it seemed to be secondary to her underlying illness rather than an inciting event. Pneumatosis intestinalis exists in both fulminant and benign forms [11], and is characterized by gas-filled cysts in the wall of either the large or small bowel. The most common and most emergent life-threatening cause of intramural bowel gas is the result of bowel necrosis [12]. Distinguishing between benign and fulminant forms of pneumatosis intestinalis remains a topic of interest, as cases of pneumatosis intestinalis with associated pneumoperitoneum have been successfully managed nonoperatively [13].

In a recent review, Greenstein et al [14] set out to identify factors that led to operative intervention and mortality. After reviewing the outcome of 40 patients with pneumatosis intestinalis, several conclusions were reached and a proposed management algorithm was introduced. Based on their findings, patients over 60 years of age, with the presence of emesis, and a WBC > 12,000 should be treated surgically. Additionally, because 70% of patients with pneumatosis intestinalis and portal venous gas have bowel ischemia [15,16], this group of patients should be treated surgically. Sepsis was found to be the only independent risk factor for mortality in patients with pneumatosis intestinalis. Based on their management algorithm, septic patients with a primary abdominal etiology should be treated surgically, while those without a primary abdominal etiology should be managed medically. Our patient clearly had an extra-abdominal source for her systemic illness and showed no evidence of infection. Based upon the above recommendations, our patient would have met criteria for medical management. As such, she recovered without operative intervention.

In summary, while TACE remains an effective and safe treatment for metastatic carcinoid not amenable to resection, this case illustrates the complexity of complications that can arise. A multi-disciplinary approach including ready access to advanced critical care facilities is recommended in managing such complex patients.

## Consent

Written informed consent was obtained from the patient for publication of this case report and accompanying images. A copy of the written consent is available for review by the Editor-in-Chief of this journal.

## Competing interests

The authors declare that they have no competing interests.

## Authors' contributions

AP was involved in the draft & finalization of manuscript and literature review. MB assisted with manuscript draft, contributed as the attending physician by providing relevant clinical information, provided interpretation of clinical information and was involved in final approval of manuscript. SS assisted with revising the manuscript critically for important intellectual content. MS assisted with revising the manuscript critically for important intellectual content. All authors read and approved the final manuscript.
